# Post-Pandemic Seroprevalence of Pandemic Influenza A (H1N1) 2009 Infection (Swine Flu) among Children <18 Years in Germany

**DOI:** 10.1371/journal.pone.0023955

**Published:** 2011-09-07

**Authors:** Rüdiger von Kries, Susanne Weiss, Gerhard Falkenhorst, Stephan Wirth, Petra Kaiser, Hans-Iko Huppertz, Tobias Tenenbaum, Horst Schroten, Andrea Streng, Johannes Liese, Sonu Shai, Tim Niehues, Hermann Girschick, Ellen Kuscher, Axel Sauerbrey, Jochen Peters, Carl Heinz Wirsing von König, Simon Rückinger, Walter Hampl, Detlef Michel, Thomas Mertens

**Affiliations:** 1 Institut für Soziale Pädiatrie und Jugendmedizin, Ludwig-Maximilians-Universität, München, Germany; 2 Abteilung für Infektionsepidemiologie, Robert Koch-Institut, Berlin, Germany; 3 Zentrum für Kinder- und Jugendmedizin, Helios Klinikum - Universität Witten/Herdecke, Wuppertal, Germany; 4 Professor-Hess-Kinderklinik, Klinikum Bremen-Mitte, Bremen, Germany; 5 Universitätskinderklinik, Universität Heidelberg, Mannheim, Germany; 6 Universitätskinderklinik, Würzburg, Germany; 7 Helios Klinikum, Krefeld, Germany; 8 Klinik für Kinder- und Jugendmedizin, Vivantes Klinikum im Friedrichshain, Berlin, Germany; 9 Helios Klinikum, Erfurt, Germany; 10 Abteilung für Kinder- und Jugendmedizin, Klinikum Dritter Orden, München-Nymphenburg, Germany; 11 Institut für Hygiene und Laboratoriumsmedizin, Helios Klinikum, Krefeld, Germany; 12 Institut für Virologie, Universitätsklinikum, Ulm, Germany; University of Hong Kong, Hong Kong

## Abstract

**Background:**

We determined antibodies to the pandemic influenza A (H1N1) 2009 virus in children to assess: the incidence of (H1N1) 2009 infections in the 2009/2010 season in Germany, the proportion of subclinical infections and to compare titers in vaccinated and infected children.

**Methodology/Principal Findings:**

Eight pediatric hospitals distributed over Germany prospectively provided sera from in- or outpatients aged 1 to 17 years from April 1^st^ to July 31^st^ 2010. Vaccination history, recall of infections and sociodemographic factors were ascertained. Antibody titers were measured with a sensitive and specific in-house hemagglutination inhibition test (HIT) and compared to age-matched sera collected during 6 months before the onset of the pandemic in Germany. We analyzed 1420 post-pandemic and 300 pre-pandemic sera. Among unvaccinated children aged 1–4 and 5–17 years the prevalence of HI titers (≥1∶10) was 27.1% (95% CI: 23.5–31.3) and 53.5% (95% CI: 50.9–56.2) compared to 1.7% and 5.5%, respectively, for pre-pandemic sera, accounting for a serologically determined incidence of influenza A (H1N1) 2009 during the season 2009/2010 of 25,4% (95% CI : 19.3–30.5) in children aged 1–4 years and 48.0% (95% CI: 42.6–52.0) in 5–17 year old children. Of children with HI titers ≥1∶10, 25.5% (95% CI: 22.5–28.8) reported no history of any infectious disease since June 2009. Among vaccinated children, 92% (95%-CI: 87.0–96.6) of the 5–17 year old but only 47.8% (95%-CI: 33.5–66.5) of the 1–4 year old children exhibited HI titers against influenza A virus (H1N1) 2009.

**Conclusion:**

Serologically determined incidence of influenza A (H1N1) 2009 infections in children indicates high infection rates with older children (5–17 years) infected twice as often as younger children. In about a quarter of the children with HI titers after the season 2009/2010 subclinical infections must be assumed. Low HI titers in young children after vaccination with the AS03_B_-adjuvanted split virion vaccine need further scrutiny.

## Introduction

In Germany, a total of approximately 223,000 laboratory-confirmed symptomatic cases of pandemic influenza A (H1N1) 2009 infections have been notified from April 1^st^ 2009 until March 31^st^ 2010, accounting for a cumulative incidence of 272 per 100,000. Extrapolation from the 78% of notifications with age information yields an incidence of 468 and 1,110 per 100,000 in age groups 1 to 4 years and 5 to 17 years, respectively. The main wave of the pandemic occurred in weeks 43 to 51 of 2009 (unpublished data, Robert Koch-Institute, Berlin, Germany).

Vaccination with a monovalent AS03_B_-adjuvanted H1N1-vaccine (Pandemrix®, GSK Pharma GmbH, Munich, Germany) was offered for persons at risk in Germany as of week 44, 2009. For adults and adolescents ≥14 years, ultimate vaccination coverage has been estimated at ∼8% on the basis of population-wide telephone surveys [1]. Data on vaccination coverage for <14 year old children are not available.

Seroprevalence studies of antibodies against A (H1N1) 2009 performed after the end of the 2009/2010 influenza season allow for a much better estimation of the cumulative incidence of pandemic A (H1N1) 2009 infections than case surveillance data if corrected for vaccination status [2]. Such data also allow to assess the current proportion of individuals with at least partial protection against A (H1N1) 2009. The proportion of subclinical infections can be estimated if a history of symptomatic infections is documented.

Seroprevalence studies in children are of particular interest since children are supposed to be the driving force of the influenza pandemic [2]–[5]. Although several seroprevalence studies of A (H1N1) 2009 antibodies have been published [6] there are few studies which include children [2], [7], [8] and only one also considering vaccination and flu-like disease history as well [7]. No study has compared antibodies against A (H1N1) 2009 after confirmed clinical disease and vaccination with pandemic vaccine in children.

We performed a multi-centre seroprevalence study on children in Germany 1) to assess the incidence of A (H1N1) 2009 infections in the 2009/2010 season, 2) to identify the proportion of subclinical A (H1N1) 2009 infections, and 3) to compare HI antibody titers in vaccinated children and in children after natural infection.

## Results

### Study population

1555 serum samples and 1511 questionnaires were collected. Fourteen hemolytic sera could not be analyzed and 52 questionnaires were excluded because essential data were missing or children were outside age limits. Merging of the remaining 1541 sera and 1459 questionnaires resulted in 1420 cases, with 373 children (26.3%) 1 to 4 years and 1047 children (73.7%) 5 to 17 years (see table 1). This corresponds to the overall distribution of these age groups in Germany (1–4: 21.2%; 5–17: 78.8%) [9]. The 8 participating hospitals were distributed nationwide.

**Table 1:** Characteristics of the post-pandemic samples.

Variable	Subcategory	Not vaccinated against A(H1N1)2009	Vaccinated against A(H1N1)2009	Unknown vaccination status against A(H1N1)2009	Total
		N = 1307	N = 101	N = 12	N = 1420
		n (column%)	n (column%)	n (column%)	n (column%)
Age	1–4 years	347 (26.6)	23 (22.8)	3 (25.0)	373 (26.3)
	5–17 years	960 (73.4)	78 (77.2)	9 (75.0)	1047 (73.7)
Sex	Male	610 (46.7)	60 (59.4)	3 (25.0)	673 (47.4)
	Female	675 (51.6)	40 (39.6)	9 (75.0)	724 (51.0)
	Missing values	22 (1.7)	1 (1.0)	0 (0.0)	23 (1.6)
(Pre-)school attendance	Yes	1097 (83.9)	89 (88.1)	8 (66.7)	1194 (84.1)
	No	201 (15.4)	11 (10.9)	4 (33.3)	216 (15.2)
	Missing values	9 (0.7)	1 (1.0)	0 (0.0)	10 (0.7)
History of infections	No infection	374 (28.6)	19 (18.8)	3 (25.0)	396 (27.9)
	> = 1 infection	914 (69.9)	78 (77.2)	9 (75.0)	1001 (70.5)
	Missing values	19 (1.5)	4 (4.0)	0 (0.0)	23 (1.6)
Diagnosis of A(H1N1) 2009	As suspected by physician	91 (7.0)	6 (5.9)	0 (0.0)	97 (6.8)
	Lab-confirmed	25 (1.9)	0 (0.0)	0 (0.0)	25 (1.8)
	No diagnosis	1030 (78.8)	80 (79.2)	10 (83.3)	1120 (78.9)
	Missing values	161 (12.3)	15 (14.9)	2 (16.7)	178 (12.5)
Seasonal vaccine*	Yes	176 (13.5)	43 (42.6)	4 (33.3)	223 (15.7)
	No	1109 (84.8)	53 (52.5)	3 (25.0)	1165 (82.0)
	Missing values	22 (1.7)	5 (4.9)	5 (41.7)	32 (2.3)

*children having been vaccinated against the seasonal influenza.

In the total sample boys and girls were evenly distributed. 84.1% of the children attended school or kindergarten. 70.5% of all children reported a history of one or more infectious disease episodes since June 2009. Influenza A (H1N1) 2009 was suspected by a physician in 97 children (6.8%) and was virologically diagnosed in 25 children (1.8%). Vaccination against seasonal influenza in 2009/2010 or in previous years was reported in 15.7% of all children. Vaccination against influenza A (H1N1) 2009 was reported for 101 of the 1420 children (7.1%).

### Hemagglutination inhibition test results - unvaccinated children

#### Pre-pandemic (control) sera

The pre- and post-pandemic HI titers and the incidence estimates for A (H1N1) 2009 infection based on the difference between post- and pre-pandemic titers are depicted in table 2.

**Table 2:** Pre-and post-pandemic hemagglutination inhibition titers (HIT) ≥1∶10 and ≥1∶40 for non-vaccinated children aged 1 to 17 years.

HIT	Age-group	Pre-pandemic controls		Post-pandemic samples		Difference: post – pre		
		n/N	(%)	n/N	(%)	(%)	95%CI Lower Limit (%)	95%CI Upper Limit (%)
> = 1∶10	1–4	2/119	1.7	94/347	27.1	**25.4**	19.3	30.5
	5–17	10/181	5.5	514/960	53.5	**48.0**	42.6	52.0
	all	12/300	4.0	607/1307	46.5	**42.4**	38.5	45.6
> = 1∶40	1–4	1/119	0.8	80/347	23.1	**22.2**	16.6	27.0
	5–17	4/181	2.2	330/960	34.4	**32.2**	27.7	35.5
	all	5/300	1.7	410/1307	31.4	**29.7**	26.4	32.4

CI = Confidence Interval.

Of the 300 control sera only 2 (1.7%) children <5 years had detectable antibodies in HIT (one child 1∶10 and the second child 1∶40). In the older age group 10 HIT-positive children were detected (5.5%) including one titer of 1∶80 (table 2). To analyze whether these pre-pandemic titers corresponded to high antibody titers against the most recent seasonal influenza A virus (A/Brisbane/59/2007(H1N1)) we tested the sera which were positive for antibodies against the pandemic A (H1N1) 2009 also for antibodies against this seasonal influenza virus. Four of the 12 children exhibiting antibodies against the pandemic A (H1N1) 2009 were negative for antibodies against the seasonal influenza A virus (geometric mean titer 1∶27). By testing additional 96 pre-pandemic sera for antibodies against the seasonal H1N1 virus we found 48.9% positive (geometric mean titer 1∶29) and no association between pandemic and seasonal H1N1 titers (Spearman correlation coefficient 0.068, p-value 0.51).

#### Post-pandemic sera

**Figure 1:**
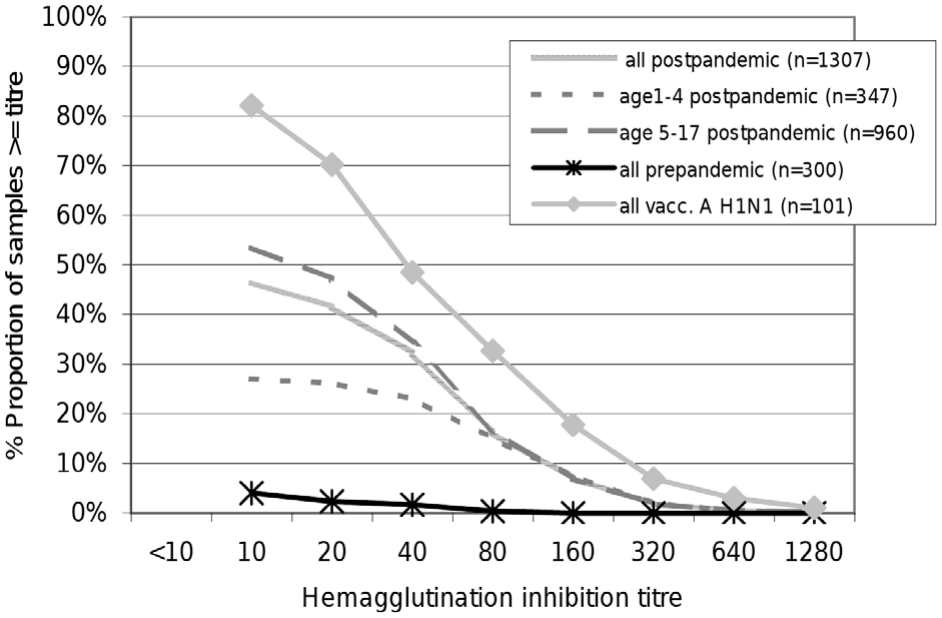
Reverse cumulative distribution curves of hemagglutination inhibition titers for antibodies against influenza A (H1N1) 2009 virus for different strata. Post-pandemic children not vaccinated against influenza A (H1N1) 2009 aged 1–17, thereof 347 aged 1–4 and 960 aged 5–17 were tested and in addition 300 pre-pandemic controls and 101 children vaccinated against influenza A (H1N1) 2009. Children aged 1–4 exhibit lower titers compared to children aged 5–17 and all post-pandemic cases. Titers of pre-pandemic children aged 1–17 are distinctly lower. Children vaccinated against A (H1N1) 2009 exhibit higher titers compared to post-pandemic children having been infected with A (H1N1).

Of 1307 children not vaccinated with Pandemrix®, HI titers of 1∶10 or above were detectable in 607 (46.5%, 95% CI: 44.3–48.8). This percentage was lower for children aged 1 to 4 years (27.1%, 95% CI: 23.5–31.3) and higher for children aged 5 to 17 years (53.5%, 95% CI: 50.9–56.2), p-value<0.0001 (figure 1 and table 2), with no difference between boys and girls.

Logistic regression revealed a higher probability of detectable HI titers by higher age and an influenza A (H1N1) 2009 infection suspected by a physician or diagnosed by virological testing. Except for a lower proportion of children with any detectable HI titers in one center (Berlin) we observed no significant regional variability. School or kindergarten attendance was not associated with HI titers.

More than 40% of the children (13.7%+30.3% = 44.0%; 95%-CI: 39.5–48.9) who had reported no infection since June 2009 had HI titers of ≥1∶10. In contrast all 25 children with virologically proven A (H1N1) 2009 infection exhibited a positive HI titer with 80% ≥1∶40. Of the children with HI titers ≥1∶10 25.5% (95% CI: 22.5–28.8) reported no history of any infectious disease since June 2009 (table 3: (91+41)/(41+95+23+5+91+198+45+20)).

**Table 3:** History of infections and hemagglutination inhibition titers (HIT) in non-vaccinated children.

		History of infections		Diagnosis of A(H1N1)2009		Cumulative missings***	Sum
HIT		No infection reported*	One or more infections**	Suspected by physician	Virologically confirmed		
<1∶10	n	168	424	21	0	87	700
	Column% (95%CI)	56.0 (51.4–60.8)	59.1 (56.2–62.2)	23.6 (17.4–32.2)	0.00 (0.2–1.13)	49.4 (43.6–55.9)	
1∶10 or 1∶20	n	41	95	23	5	33	197
	Column% (95%CI)	13.7 (10.8–17.4)	13.2 (11.3–15.5)	25.8 (19.3–34.6)	20.0 (11.0–37.5)	18.8 (14.6–24.3)	
> = 1∶40	n	91	198	45	20	56	410
	Column% (95%CI)	30.3 (26.3–35.0)	27.6 (25.0–30.5)	50.6 (42.5–59.7)	80.0 (67.0–91.8)	31.8 (26.6–38.1)	
Total		300	717	89	25	176****	1307

*“Infection” was defined as any infection, since the clinical picture of flu in children may not be confined to typical symptoms of upper or lower respiratory tract infection.

**without suspected or confirmed diagnosis of A (H1N1) 2009.

***Cumulative missing values in “history of infections” or/and “diagnosis of A (H1N1) 2009”.

****There were 161 children with missing values for the variable “diagnosis of A (H1N1)” (no such diagnosis; suspected by physician; lab-confirmed) and an additional 19 children with missing values in “history of infections” (no infection; one or more infection) (see table 1). Four children had missing values regarding both variables, therefore the total of missing values was 176 (161+19−4 = 176).

### Hemagglutination inhibition test results – vaccinated children and children after laboratory-confirmed influenza A (H1N1) 2009 infection

**Figure 2:**
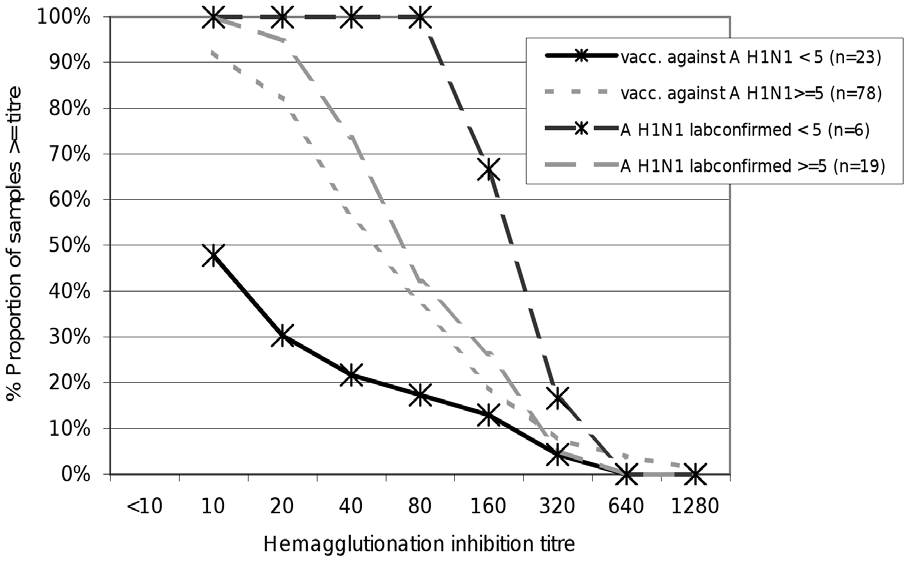
Reverse cumulative distribution curves of children vaccinated against the A (H1N1) 2009 virus <5 years (n = 23), > = 5 years (n = 78) and in children with laboratory-confirmed A (H1N1) 2009 infection <5 years (n = 6), > = 5 years (n = 19). Vaccinated children and children with laboratory-confirmed A (H1N1) infection were mutually exclusive. Children vaccinated against the A (H1N1) 2009 virus exhibit lower titers compared to children with laboratory-confirmed A (H1N1) 2009 infection. This can be observed in both age groups with greater difference in younger children.

All 6 children 1 to 4 years old (100%, 95% CI: 61.0–100) with a history of laboratory-confirmed A (H1N1) 2009 infection exhibited HI titers of 1∶80 or above (dashed dark line in figure 2), but only 11 of 23 children in this age group (47.8%, 95% CI: 33.5–66.5) who reported vaccination with Pandemrix® had HI titers ≥1∶10, and 21.7% (95% CI: 12.0–40.4) had HI titers ≥1∶40 (dark line). The differences between HI titers in children aged 1 to 4 years after natural infection and after vaccination were statistically significant (Kolmogorov-Smirnov test for difference between two curves, p-value: 0.003). For children aged 5 to 17 years no such statistically significant difference (grey dashed line = natural infection; dotted line = vaccinated) was observed (Kolmogorov-Smirnov test, p-value: 0.752).

In addition antibody titers in children vaccinated with Pandemrix® aged 1 to 4 years (dark black line) were significantly lower than in vaccinated children aged 5 to 17 years (grey dotted line) (Kolmogorov-Smirnov test, p-value: 0.0002). In contrast, after natural infection higher titers were observed in the younger children as compared to the older age group. The numbers, however, are small and the difference was not statistically significant (Kolmogorov-Smirnov test, p-value: 0.0941).

There appeared to be an inverse association between the geometric mean HI titers against the A (H1N1) 2009 influenza virus and previous vaccination with seasonal influenza vaccine. In children vaccinated both with the seasonal and the pandemic vaccine the OR for HI titers ≥1∶40 was 0.68; 95% CI 0.46–1.01) compared to children vaccinated with the pandemic vaccine alone.

## Discussion

Comparison of seroprevalence against influenza A (H1N1) 2009 in pre- and post-pandemic sera indicates that about a quarter of the unvaccinated preschool (1–4 years) and almost half of the unvaccinated school age children (5–17 years) were infected with influenza A (H1N1) 2009 during the pandemic in Germany. A titer of ≥1∶40, usually considered “protective” (i.e. associated with >50% reduction of the risk of contracting influenza) [10], was observed in 23% of preschool and 36% of school age children, taken together vaccinated and unvaccinated (figure 1). A history of pandemic influenza vaccination was reported for ∼7% of participating children, a percentage similar to the existing estimate of ∼8% for persons >14 years in Germany [11]. HI titers ≥1∶40 in as little as 22% of vaccinated children aged 1 to 4 years approximately 4–8 months after vaccination point to a need for further evaluation of the immune response to adjuvanted influenza vaccines in young children although there is a possibility that this is due to chance given that there were only 23 vaccinated children and 6 children with lab-confirmed A (H1N1) 2009 infection <5 years.

The incidence of A (H1N1) 2009 infections in children and adolescents in Germany was similar to those reported from the UK (after first wave) [2] and New Zealand [7], but somewhat lower than after the second wave in UK [8]. As in these studies, we found a considerably higher incidence of infections in school age compared to preschool children, which also corresponds to the reported age-specific incidence of infections from surveillance data in Germany (unpublished data of the Robert Koch-Institute).

Approximately 25% of children with detectable antibodies against A (H1N1) 2009 reported no history of any infectious disease episode during the pandemic influenza season. Disease history in our study is based on parents' or participants' recollection over a period of several months and may therefore not be fully reliable. It is likely that mild events of disease may not have been recalled which would result in an overestimation of subclinical infections. On the other hand, reported infection episodes may have been unrelated to influenza A (H1N1) 2009, which means the proportion of subclinical A (H1N1) 2009 infections could have been even higher. In the study from New Zealand [7], the proportion of subclinical infections was estimated as 45% (for all ages). Smaller studies among contact persons of confirmed cases found proportions of subclinical infections between 9% and 73% [12]–[14].

In Germany the pandemic vaccine became gradually available starting from the last week of October 2009. While two doses had been recommended initially, the official vaccination recommendation for children (as well as for adults) was changed from a two dose to a single dose regimen as of December 14th 2009 [15]. The vaccine used was an AS03B-adjuvanted split virus vaccine (3.75 µg hemagglutinin) with a half dose recommended for children aged 6 months to 9 years. The lower antibody titers observed in our study in young compared to older children after vaccination are unlikely to be explained by chance. Differential misclassification of the vaccination history against the pandemic influenza virus by the children's age is unlikely. Although we have not ascertained the number of vaccine doses, most children had presumably received one dose only, because by the time the second dose was due (≥3 weeks after the first dose), a second dose was no longer recommended. It remains unclear whether this vaccination regimen is the reason for the lower antibody titers in the young children. Concern regarding a possibly weaker immune response in young children has also been raised for the non-adjuvanted pandemic influenza vaccines [3], [16]. Available evidence suggests high seroconversion rates in young children after two doses of AS03B-adjuvanted split virus vaccine (UK) [17] and possibly also after one dose (Spain) [18].

Clinical vaccine effectiveness, however, has been demonstrated for one dose of the vaccine in persons ≥14 years and most recently for children as well [19]–[21]. In young children, however, vaccine effectiveness appeared to be lower than in adolescents and the 95% CI was wide [21].

For children who had been vaccinated with a seasonal vaccine in previous years, titers after vaccination with the pandemic vaccine were somewhat, but not significantly lower. A similar finding was recently observed in a vaccine effectiveness study of the AS03 adjuvanted influenza A (H1N1) 2009 vaccine for adults [22], [23].

This seroprevalence study was performed with an optimized in-house hemagglutination inhibition test which gave clear and highly reproducible results and allowed to determine titers of 1∶10 against influenza virus A (H1N1) 2009. We decided not to use a micro neutralization test in addition since the HIT is widely accepted as reference test for detection of subtype-specific influenza antibodies and the inter-assay and inter-laboratory reproducibility and standardization is better for HIT than for micro neutralization ([24], [25] and unpublished own data). Furthermore it has been shown in multiple studies that there is a good overall correlation between the results of the two assays for H1N1 [2], [8], [24].

Instead we optimized the HIT-SOP to reach a high specificity without loosing relevant sensitivity. We found much fewer HIT positive results in the pre-pandemic sera than other studies, where arbitrary cutoffs of 1∶32 or 1∶40 had to be introduced [2], [24], [26], [27], [28]. We detected only 12 of the 300 control sera repeatedly positive for A (H1N1) 2009 influenza virus antibodies and only 2 in the younger age group of 1 to 4 years, while in the study group all children with virologically proven pandemic A (H1N1) 2009 infection exhibited mainly high antibody titers. Additionally, in a subsample of the control and study sera no correlation between antibodies against seasonal A/Brisbane/59/2007(H1N1) and antibodies against A (H1N1) 2009 influenza was found [28]. Therefore a simple cross reactivity at least between the antibodies against these two H1N1 influenza viruses can be excluded [27]. The prevalence of antibodies against seasonal A H3N2 was very low in sera from the study and control group (data not shown).

The external reference serum pool obtained from the National Institute for Biological Standards and Control (NIBSC), with a defined titer after multi-laboratory testing as 1∶180, however, absolutely reproducibly yielded a titer of 1∶80 (i.e. practically one twofold dilution lower) in our test. Although the titers determined by our method are slightly lower (one twofold dilution) than determined by other HIT-SOPs, we did not change our method because we had no indication for false negative results and we wanted to minimize false positive results.

In order to allow for a valid estimation of the incidence of A (H1N1) 2009 during the 2009/2010 season we accepted only pre-pandemic sera that were collected within 6 month before onset of the A (H1N1) 2009 pandemic in Germany, thereby assuring that the seasonal influenza virus A experience was as similar as possible in both groups and that the relevance of waning antibody titers was minimized. Within the age groups 1 to 4 and 5 to 17 years the numbers of pre- and post-pandemic sera were collected proportionally by age in order to assure structural homogeneity.

Serum samples from children attending medical care for seroprevalence studies on pandemic influenza, as also used in a recent study from the UK [2], may not be fully representative. A population-based study in children with possibly higher representativity, however, might be fraught with selection bias in case families with a presumed history of influenza A infection were more likely to participate.

In conclusion, serologically determined incidence of influenza A (H1N1) 2009 infections in children indicates overall high infection rates, with older children (5–17 years) infected twice as often as children between 1 and 4 years. In about a quarter of the children with HI titers after the season 2009/2010 subclinical infections must be assumed. Our study provides the first estimate of post-pandemic population partial immunity in this age group in Germany. The observed low HI titers in young children after vaccination with the AS03_B_-adjuvanted split virion vaccine need further scrutiny.

## Methods

### Serum and data collection

Eight German pediatric primary care hospitals (Bremen, Berlin, Krefeld, Wuppertal, Erfurt, Würzburg, Mannheim, München) provided sera from April 1^st^ to July 31^st^ 2010 obtained during blood withdrawal for routine laboratory testing from in- or outpatients aged 1 to 17 years. Children with an illness impeding an adequate immune response were excluded, so were children with serious conditions. Eligible children and their parents were informed about the study with age-adapted study descriptions and asked for their written consent. The study was approved by the Ethics Committee of the Bavarian Medical Association.

The following data were collected on a one-page questionnaire filled in by the parents: age and sex of the child, history of infectious disease episodes since June 2009, history of “swine flu” by physician's tentative clinical diagnosis vs. laboratory-confirmed diagnosis, history of pandemic or seasonal influenza vaccination, and kindergarten/school attendance. Questionnaires were sent to the study office in Munich and double-entered into a common data base.

Minimum 400 µl serum samples were sent by cool pack to the virological study laboratory in Ulm for determination of antibody titers. Questionnaires and serum samples were labeled with corresponding anonymous serial numbers. Laboratory staff was not aware of the data collected on the serum donors. Serological results and questionnaire data were linked after the end of the serological testing only.

### Pre-pandemic samples

300 control sera for the study were taken from the frozen stored sera bank of the virological institute in Ulm, originally sent for routine virological diagnostic. The sera were selected according to two criteria: 1) the respective blood samples had to be collected between November 2008 and April 2009 and 2) for the age groups 1 to 4 and 5 to 17 years the numbers of pre-pandemic sera were proportionally to the age distribution of sera in the post-pandemic study sample.

### Laboratory procedures

For determination of the antibody responses an in-house hemagglutination inhibition test (HIT) was established in Ulm to minimize non-A (H1N1) 2009- specific results [11], [24], [29]. During establishment of the HIT different buffers, protein additions, and erythrocytes from different species in different concentrations and receptor destroying enzymes (RDE) were evaluated [26].

The study and control sera were tested using the optimized HIT standard operation procedure (SOP). To destroy non-specific inhibitors of influenza virus hemagglutination sera were pretreated with RDE (DENKA Seiken CO Ltd, Tokyo, Japan) [26]. RDE was reconstituted with H_2_O and stored frozen in aliquots (100 µl). Before usage RDE stock solution was diluted 1∶20 in 0.9% NaCl. 50 µl Serum was mixed with 100 µl RDE and incubated 16–18 h at 37°C. After inactivation 30 min at 56°C, 350 µl hepes saline albumine (HSA, 0.14 M NaCl, 0.025 M hepes, 0.001 M CaCl2+1%BSA (before usage), pH 6.2–6.3) was added (1∶10 serum dilution). Two fold serial dilutions (25 µl) of sera were performed using HSA+1% BSA in microtiter plates (round bottom). Influenza-A virus antigens (pandemic A/California/7/2009 (H1N1), seasonal A/Uruguay/716/2007 (H3N2), seasonal A/Brisbane/59/2007 (H1N1), all GlaxoSmithKline, Dresden, Germany) were diluted after HI pre-testing to obtain a solution containing 4 hemagglutinating units (HU). After addition of 25 µl antigen, shaking, and incubation (30 min, room temperature (RT)) 50 µl newborn chicken erythrocytes (Laboratory Dr. Merk&Kollegen, Ochsenhausen, Germany) (0.5% in HSA) were added, plates shaken again, incubated (2 h, RT) and read by 2 independent evaluators using a magnification lens (×10). The titer is defined as the highest serum dilution showing complete inhibition of the agglutination of erythrocytes. A serum control for every serum without addition of antigen and positive (1∶80), low positive (1∶10) and negative (1∶<10) control sera were included for every test. An external reference serum pool was obtained from the Health Protection Agency National Institute for Biological Standards and Control (NIBSC), UK, and used to validate our internal positive control sera. Test results were accepted if the back titration of antigen revealed 4 HU and the control sera yielded correct results. Sera showing non-specific hemagglutination were tested again after absorption (1 h at 4°C) with 50 µl of 40% newborn chicken erythrocyte suspension in HSA. For determination of sensitivity and specificity of our HIT we used the 300 pre-pandemic sera (true negative) and 47 sera available from our diagnostic laboratory at that time including 11 Ulm patients with virologically proven pandemic A (H1N1) 2009 infection and 36 vaccinated co-workers (true positive). Sensitivity was 1.00 and specificity 0.96 for titer ≥1∶10. With 14 hemolytic study sera HI titer could not be determined.

### Statistical analysis

Reverse cumulative distribution curves were calculated to compare proportions above different threshold hemagglutination inhibition titers for unvaccinated children (by age groups 1–4 years and 5–17 years and for children with laboratory-confirmed A (H1N1) 2009 infection), for children vaccinated against A (H1N1) 2009 and for controls.

Logistic regression models were used to examine differences in HI titers according to age, sex, region, history of infections since June 2009, vaccination against seasonal influenza and kindergarten/school attendance. Chi-square and Kolmogorov-Smirnov tests were used to compare HI titers between different strata of children.

To estimate the incidence of A (H1N1) 2009 infection during the 2009/2010 pandemic in Germany we calculated the difference between the proportion of sera with a HIT titer >1∶10 in pre- and post-pandemic samples. Additional evaluations were done using an arbitrary cut-off of ≥1∶40 titer, generally assumed to indicate protection. Confidence intervals for the difference in proportion of children achieving titers ≥1∶10 and ≥1∶40 pre-and post-pandemic were calculated using the Newcombe-Wilson method [30].

All statistical analyses were done with SAS 9.2 and Excel 2003.
